# Bidirectional biomimetic flow sensing with antiparallel and curved artificial hair sensors

**DOI:** 10.3762/bjnano.10.4

**Published:** 2019-01-03

**Authors:** Claudio Abels, Antonio Qualtieri, Toni Lober, Alessandro Mariotti, Lily D Chambers, Massimo De Vittorio, William M Megill, Francesco Rizzi

**Affiliations:** 1 Center for Biomolecular Nanotechnologies @UNILE, Istituto Italiano di Tecnologia, Arnesano (LE), I-73010, Italy; 2 Rhine-Waal University of Applied Sciences, Faculty of Technology and Bionics, Kleve, D-47533, Germany; 3 Università del Salento, Dipartimento di Ingegneria dell’Innovazione, Lecce (LE), I-73100, Italy; 4 Westphalian University of Applied Sciences, Department of Mechanical Engineering, Bocholt, D-46397, Germany; 5 Università di Pisa, Dipartimento di Ingegneria Civile e Industriale, Pisa, I-56122, Italy

**Keywords:** artificial hair sensor, biomimetics, flow direction, flow sensing, robotics

## Abstract

**Background:** Flow stimuli in the natural world are varied and contain a wide variety of directional information. Nature has developed morphological polarity and bidirectional arrangements for flow sensing to filter the incoming stimuli. Inspired by the neuromasts found in the lateral line of fish, we present a novel flow sensor design based on two curved cantilevers with bending orientation antiparallel to each other. Antiparallel cantilever pairs were designed, fabricated and compared to a single cantilever based hair sensor in terms of sensitivity to temperature changes and their response to changes in relative air flow direction.

**Results:** In bidirectional air flow, antiparallel cantilever pairs exhibit an axially symmetrical sensitivity between 40 μV/(m s^−1^) for the lower air flow velocity range (between ±10–20 m s^−1^) and 80 μV/(m s^−1^) for a higher air flow velocity range (between ±20–32 m s^−1^). The antiparallel cantilever design improves directional sensitivity and provides a sinusoidal response to flow angle. In forward flow, the single sensor reaches its saturation limitation, flattening at 67% of the ideal sinusoidal curve which is earlier than the antiparallel cantilevers at 75%. The antiparallel artificial hair sensor better compensates for temperature changes than the single sensor.

**Conclusion:** This work demonstrated the successive improvement of the bidirectional sensitivity, that is, improved temperature compensation, decreased noise generation and symmetrical response behaviour. In the antiparallel configuration, one of the two cantilevers always extends out into the free stream flow, remaining sensitive to directional flow and preserving a sensitivity to further flow stimuli.

## Introduction

### Biological lateral line organ

**Figure 1:**
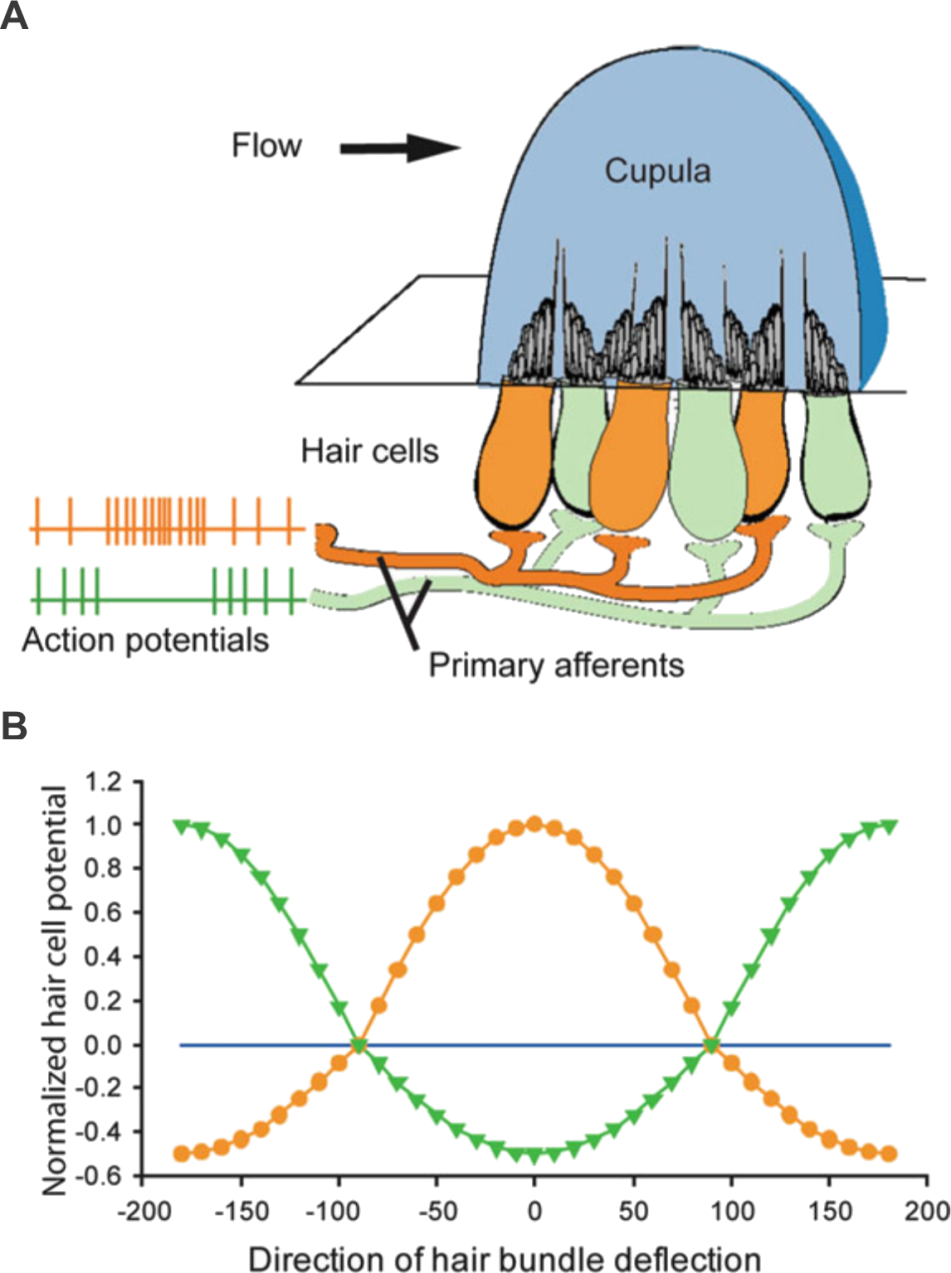
Opponent organization of lateral line neuromasts. (A) Hair cells on any given neuromast are oriented in one of two opposing (orange and green) directions, resulting in a single axis of best sensitivity. Modified with permission from [13], copyright 2004 John Wiley and Sons. (B) Hair cell responsiveness, modeled as a cosine function of the direction of hair bundle deflection for the two oppositely oriented populations of hair cells. Reproduced with permission from [14], copyright 2014 Springer Nature.

Flow sensors in nature often have a morphological polarity, such as the hair cell sensors in the lateral line of fish [1], in jellyfish [2], arthropods [3–4] and crickets [5–8], as well as the hair cells in audition of humans [9]. The lateral line of a fish is an intricate flow sensing network of individual sensors, called neuromasts, which are located on the surface and subsurface on the body of the fish. Over millions of years, two different types of neuromasts have evolved, canal and superficial neuromasts, which encode the pressure gradient over the body’s surface [10] and velocity of the flow [11], respectively. Whereas superficial neuromasts are located at the outside of the skin being in direct contact to the water, canal neuromasts form a part of an internal canal system located beneath the skin in which water streams in by entering external openings. Although canal and superficial neuromasts vary in their anatomical structure, both neuromast types are similar in their functional principle: water flows into the canal or around the skin and bends a jellylike cupula protruded into the fluid. Canal neuromast cupulae are typically hemispherical with a diameter in the hundreds of micrometers, whereas the much smaller superficial neuromast cupulae are bullet-shaped and typically around 50–100 μm in height and 10 μm in width [12]. Figure 1 illustrates the anatomical structure of the neuromast.

**Figure 2:**
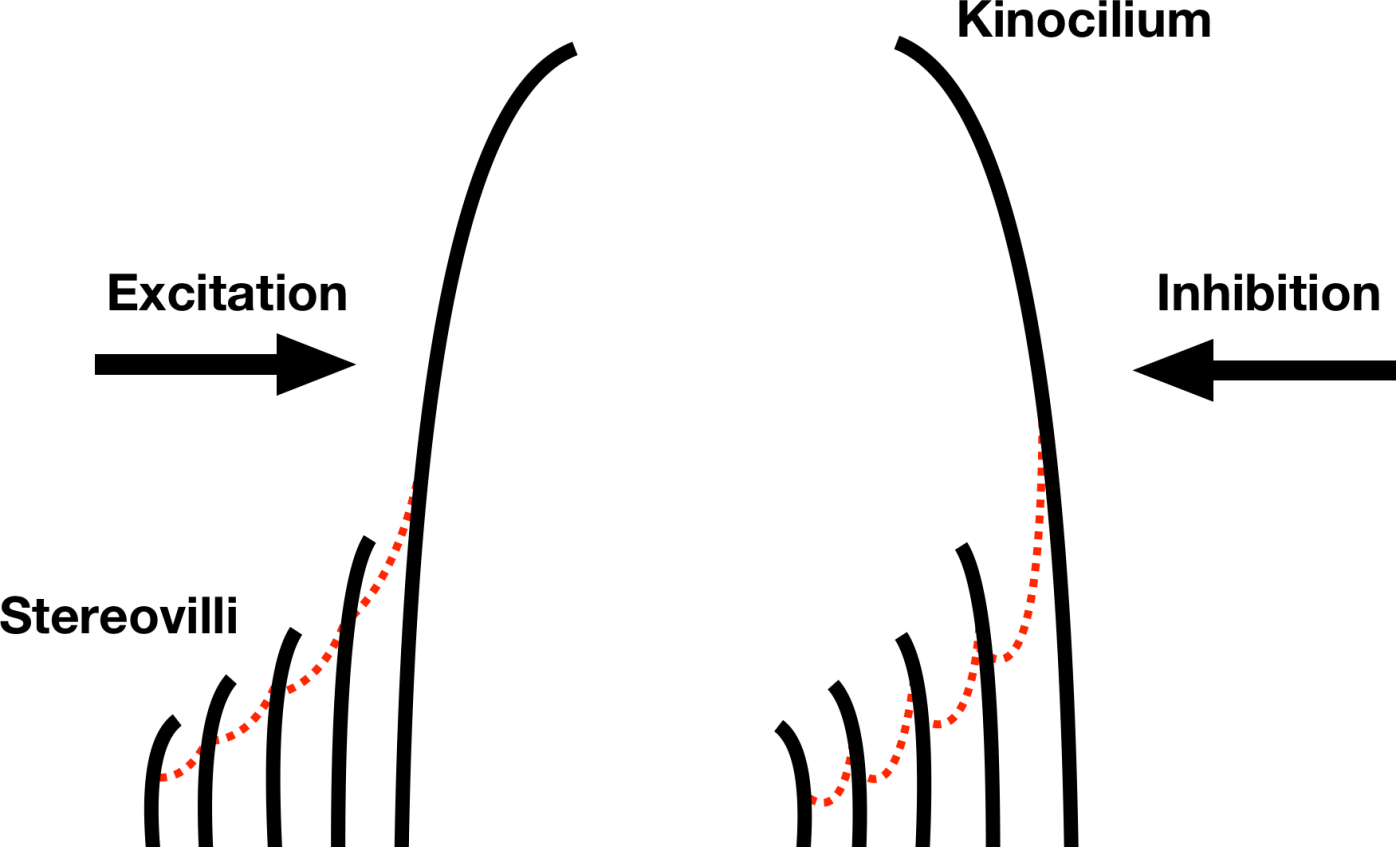
Simplified 2D model of a neuromast hair cell bundle of fish: stereovilli increase in stepped length up to the tallest kinocilium. Stereovilli and kinocilium are connected by tip links (red dotted line). Mechanical work (bending) stretches the tip links and pulls ion channels open. The channel opening causes a change of cell potential which in turn triggers an action potential. Mechanical deflection of the kinocilium away from the stereovilli causes excitatory response and vice versa (toward stereovilli) causes inhibitory response.

Neuromasts contain bundles of hair cell stereovilli which are deflected mechanically by a flow stimulus which triggers a membrane potential shift. The neuromast has a directional sensitivity which is determined by an axis of orientation of the stereovilli and kinocilium of the individual hair cells. The stereovilli increase in stepped length up to the tallest kinocilium, as shown in Figure 2. This morphology as well as the presence and arrangement of the various tip links between the stereovilli and the kinocilium generate a directional sensitivity and defines a best sensing direction. Indeed, as the flow bends the graded-height stereovilli, it deflects the cilia, allowing the mechanoreception of the flow stimulus to be transduced as a depolarisation in the membrane, as pictured in Figure 2. The opposing flow pushes the kinocilium towards the stereovilli, restricting their movement, relaxing the linkages and thus inhibiting the corresponding action potential [9]. Therefore, depending on the flow mechanical excitation direction, hair cells within the same neuromast assume a bending orientation that is antiparallel (i.e., opposite orientation) to each other, thus generating an axis of sensitivity or best direction.

Depolarising events are associated with decreasing membrane electrical resistance while hyperpolarization responses with an increase in membrane electrical resistance [15]. A new flow stimulus which occurs on top of already existing flow is difficult to decouple using one sensor orientation. This is overcome in nature so that an oscillatory flow imposed in addition to a steady flow can be sensed using two sensory cells with opposite excitatory stimulus best directions [16]. The fish lateral line then further deploys these sensors to decouple multiple flow stimuli by using an array of neuromast sensors [17]. Another key feature of the directional sensitivity is that if the incoming flow stimulus is tested at varying angles to the biological hair cell, the response, as measured on the afferent nerve, is cosine-like in relation to the stimulus angle [1], as illustrated in Figure 1.

The signal detection capability of superficial neuromasts strongly depends on the hydrodynamics of the boundary layer and the mechanical properties of the cupula [11]. Caused by its viscosity, water close to a body’s surface moves slower than the flow stimulus, which creates a spatial gradient of flow velocity in the boundary layer. The boundary layer thickness is commonly defined as the distance from a surface where the velocity is nearly equal to the free stream velocity. It increases with distance from the leading edge and with decreasing flow velocity [18]. In the theoretical approach described by McHenry et al. [11], the deflection of the cupula structure in flow was modelled by considering the mechanical contributions of the boundary layer, the components of the cupula morphology and the fluid-structure interaction. By treating the cupula structure as a cylindrical beam, the forces generated by the flow upon and within the cupula were calculated and cupula deflection was described as a function of its height above the skin [11].

### Artificial hair sensors

As comprehensively described in several literature reviews [19–24], different flow sensing approaches and design methodologies for producing hair cell-like flow sensors were demonstrated in various laboratories world-wide, such as thermal transfer [25], pressure distribution [26], torque transfer [27], mechanical bending [28], and optical detection principles [29–30]. Commonly used electrical measurement procedures in hair cell-like sensors depend on capacitive or piezo-resistive approaches.

The first attempts have been made by designing and developing hot-wire anemometry (HWA) sensors [31]. The HWA principle is based on a fluid flowing over a heated wire which causes heat loss due to convective currents [25]. As the resistance of the wire depends on its temperature, the cooling rate gives information about the flow velocity. Researchers from the Micro and Nanotechnology Laboratory of the University of Illinois at Urbana-Champaign, Chen and Liu [32], developed a new type of flow sensor based on the HWA principle, realised by using an efficient microfabrication process which combines surface micromachining and a 3D assembly method [28]. Two support beams, each being 2.7 μm thick, elevate the thermal wire out of the plane up to only 1 mm, hence reducing interference to the flow. The thermal wire is made of platinum or tungsten and its length varies between 50 and 200 μm. This approach based on their novel 3D assembly method makes it possible to form large arrays of sensors on a variety of substrate materials.

The research group from the MESA+ Institute for Nanotechnology at the University of Twente developed a capacitive artificial neuromast based sensor platform that was integrated as high-density arrays to measure high-frequency acoustic flow patterns based on drag force [33–36]. The artificial neuromast is made of a vertical pillar with heights between 400 and 1000 μm, heights similar to the sensory hairs found on crickets. To distinguish between rotation and translation normal to the substrate, two electrodes on a membrane were attached to the base of the pillar. The capacitive read-out indicates the movement of the pillar. High-density arrays were designed to increase the total capacitance value and thereby the overall sensitivity. Successfully carried out measurements (capacitance vs voltage, frequency dependence and directional sensitivity) demonstrated the viability of the capacitive-based flow sensing approach. With a pillar geometry of 900 μm × 50 μm and a two-dimensional sensor separation of ≈250 μm, the flow sensor array described by Bruinink et al. [35] achieved very high sensitivity (minimal detectable flow amplitude) down to 2 mm s^−1^ in air flow. The 100-fold increase in acoustic sensitivity in comparison to their first-generation capacitive-based flow-sensor arrays [33–34] was mainly achieved by an increase in pillar length from 450 to 900 μm, a decrease in diameter from 50 to 25 μm and the removal of the membrane curvature.

Table 1 gives an overview of bent piezoresistive cantilever structures, a subset of closely related MEMS flow sensors for direct comparison in this study. A more comprehensive list of previously described piezoresistive based artificial hair sensors, including geometries such as vertical beams, vertical pillars and bent flags, can be found in [37].

**Table 1:** Previously described, bent-cantilever-based flow sensors ordered by year of publication. Comparative overview of cantilever geometries and flow velocity related performance indicators (sensitivity *S*, measurement range *R*). Hair geometry defined as product of length × width (or diameter) × thickness. Adapted from [37].

Authors	Geometry	Performance

Wang et al. (2007) [38]	bent cantilever (4000 μm × 400 μm × 1 μm)	*S*_air_ = 0.0284 Ω/(m s^−1^); *R*_air_ = 0–45 m s^−1^
Wang et al. (2008) [39]	bent cantilever (4450 μm × 200 μm × 20 μm)	n/a
Du et al. (2009) [40–41]	cantilever (500 μm × 500 μm × 10 μm)	*S*_air_ = 60 μV/(m s^−1^)
Zhou et al. (2009) [42] Zhang et al. (2010) [43]	bent cantilever (100 μm × 20 μm × 1 μm)	*S*_water_ = 1.5–3.5 Ω/(cm s^−1^); *R*_water_ = 0–0.23 m s^−1^
Qualtieri et al. (2011) [44]	bent cantilever (600 μm × 100 μm × 0.7 μm)	n/a
Qualtieri et al. (2012) [45]	bent cantilever (1500 μm × 100 μm × 4 μm)	*S*_water_ = 0.7 mV/(cm s^−1^); *R*_water_ = 0.05–0.35 m s^−1^

To systematically investigate the response behaviour of their piezoresistive cantilever-based air flow sensor, Wang et al. [38] performed wind tunnel tests with three different cantilever beam lengths (400 μm, 1200 μm and 2000 μm) at air flow velocities ranging between 0 and ≈45 m s^−1^. Progressively increasing the air flow velocity increased resistance signals approximately linearly. Average sensitivities for the individual cantilever beam lengths were found to be 0.0134, 0.0227 and 0.0284 Ω/(m s^−1^), respectively. Aiming to detect air flow direction, Wang et al. [39] positioned four microcantilever beams (4000 μm long and 400 μm wide) perpendicular to each other. While air propagated through the sensor array in parallel to two opposing, bent beams, air flow direction was determined by measuring the variation of platinum resistance between different cantilever beams with an external LCR meter (inductance *L*, capacitance *C*, and resistance *R*). The least resistance variation was caused by the upwind cantilever, whereas the largest resistance variation was found for the downwind cantilever. In contrast, the resistance variations of the two perpendicular cantilever beams were almost equal to zero.

Du et al. [40] connected a rectangular plate (3000 μm × 2500 μm × 10 μm) to two squared cantilevers (500 μm × 500 μm × 10 μm) which bent as air flow hit the plate. While the plate received the drag force of the air flow, the cantilevers measured the drag force using platinum strain gauges. One variable resistor (strain gauge) on each of the two cantilevers and two fixed resistances on the sensor substrate were used in a Wheatstone bridge circuit, which converted the resistance change into a change in output voltage that decreased in forward and increased in backward direction with air flow velocities ranging between −18 m s^−1^ and 18 m s^−1^. The flow sensor demonstrated a sensitivity of 60 μV/(m s^−1^) at around 18 m s^−1^. By attaching two flow sensor units perpendicular to each other, the sensor demonstrated sensitivity to multidirectional air flow with a maximal error of 9.2% [41].

Zhang et al. [43] calibrated their bent piezoresistive flow sensors in deionized water with different cantilever beam lengths (100 μm, 200 μm and 400 μm) at varying water flow rates between 0 and 0.2 m s^−1^. The microcantilevers were able to measure small flow rates between 0 and 0.23 m s^−1^, with a sensitivity ranging between 1.5 and 3.5 Ω/(cm s^−1^).

Qualtieri et al. [44] developed bent artificial hair sensors (600 μm × 100 μm × 0.7 μm) that demonstrated bidirectional sensitivity to nitrogen flow. Equipped with a 80 μm long nickel–chrome (80/20) piezoresistor (strain gauge), the artificial hair sensor was sensitive to nitrogen flow along both cantilever directions. However, the strain gauge resistance revealed an asymmetrical response behaviour. While the piezoresistance varied by around 0.44% in the forward flow direction (cantilever flattened), a curled-up cantilever varied only by 0.07% in the backward direction. Extending the cantilever and strain gauge length, Qualtieri et al. [45] characterized artificial hair sensors (1500 μm cantilever beam length) in water at flow velocities up to 0.5 m s^−1^. It was shown that the sensitivity of the flow sensor to a specific dynamic range can be tuned by choosing the thickness of a thin, waterproof parylene layer accordingly [46]. A parylene coating of 0.5 μm thickness showed strain-hardening behaviour with a linear sensitivity of ≈0.2 V/(m s^−1^) at water flow velocities lower than 0.2 m s^−1^. In comparison, a flow sensor coated with 2 μm parylene showed strain-softening behaviour with a linear sensitivity of ≈0.07 V/(m s^−1^) at higher flow velocities between 0.25 m s^−1^ and 0.35 m s^−1^. Signal saturation in air was obtained at about 40 m s^−1^ [47].

### Antiparallel cantilever design

**Figure 3:**
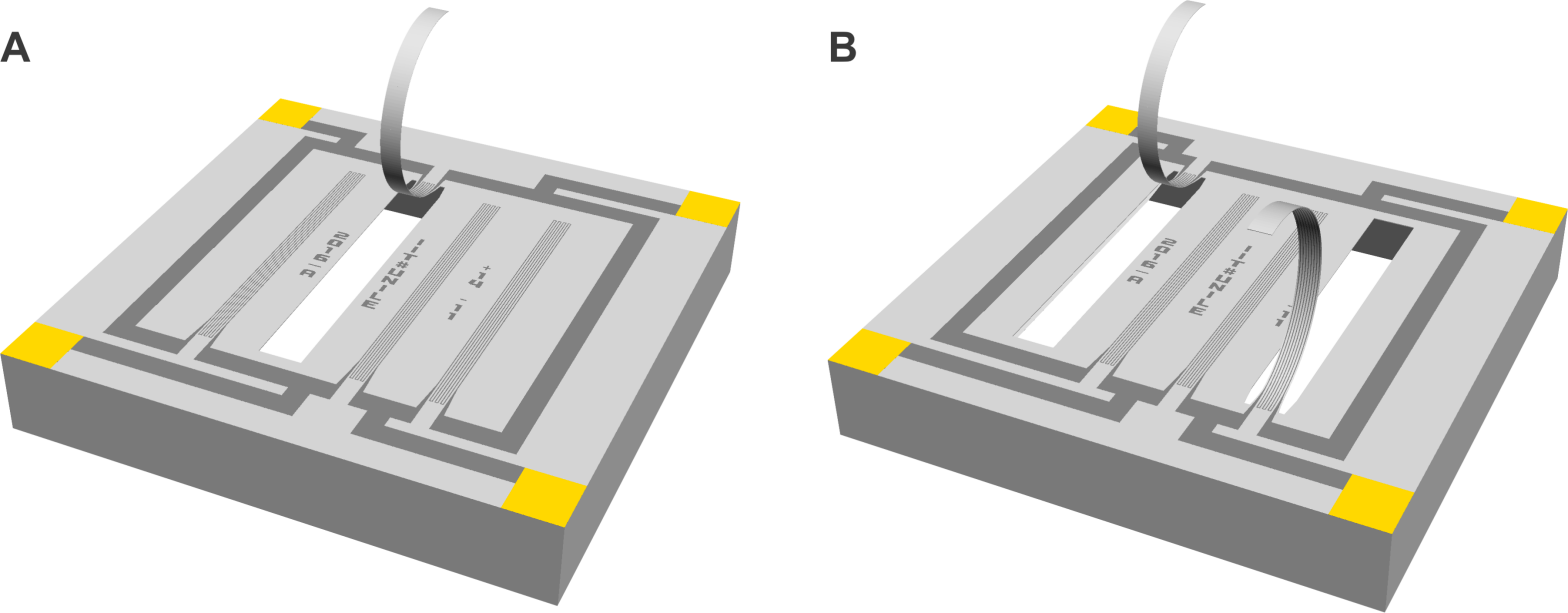
CAD models of the single and antiparallel hair flow sensor with strain gauges bent out-of-plane. (A) Single strain gauge forms a Wheatstone quarter-bridge, whereas (B) antiparallel strain gauges form a Wheatstone half-bridge configuration.

In this study, keeping the same cantilever dimensions as described by Qualtieri et al. [45], we expanded on our work to optimize the sensor response to flow by using an antiparallel configuration. Antiparallel cantilevers are fabricated and fully characterized which are coupled as a sensor with bidirectional sensitivity. The novel sensor design and arrangement is calibrated and stimulated by air flow in multiple directions. The effects of temperature change were measured. To compare the antiparallel cantilever with a single cantilever design, a single hair sensor reference model with equal cantilever dimensions and material composition was designed, manufactured and investigated in the presented study, as shown in Figure 3. The main difference between a single and two antiparallel cantilevers is that one variable resistor (sensing strain gauge) forms a quarter-bridge configuration (in the case of the single cantilever), whereas two represent a half-bridge configuration (antiparallel cantilevers) in the Wheatstone bridge circuit.

Flow velocities ranging between 10 m s^−1^ and 32 m s^−1^ were used to characterize the two sensor variants in air. The Reynolds equivalence with regard to speed in water is ≈1/15 that in air with kinematic viscosities of ν ≈ 1 × 10^−6^ m^2^ s^−1^ for water and ≈15 × 10^−6^ m^2^ s^−1^ for air at 20 °C [18]. That is, 10–30 m s^−1^ flow speeds in air are (Reynolds) equivalent to ≈0.7–2 m s^−1^ in water, which happen regularly in a biological context. A 35 cm trout, for instance, swims with a speed of up to 3.5 m s^−1^ (10 body lengths per second) [48].

Reynolds numbers for the cantilever were roughly estimated based on the assumption of ideal laminar air flow conditions, following the calculation presented in [49]. Reynolds numbers range between *R*_e_ ≈ 67 (for 10 m s^−1^) and *R*_e_ ≈ 213 (for 32 m s^−1^) for air at 20 °C (kinematic viscosity ν = 15.06 × 10^−6^ m^2^ s^−1^) and a cantilever beam width *d* = 100 μm (characteristic length). This suggests steady flow conditions in the wind tunnel at the cantilever tip. In laminar flow, the boundary layer thickness δ over a flat plate is approximately δ ≈ 4.91 
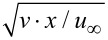
 with kinematic viscosity ν, distance *x* along the surface from the leading edge, and free stream velocity *u*_∞_ [18]. For instance, given an air flow of 30 m s^−1^ over a 30 cm sized object, the boundary layer would be approximately 1.9 mm thick. The presented flow sensors, inspired by the mechanoreceptive neuromasts found in fish, are adequate for measuring phenomena that take place in the boundary layer, as the total height of the flow sensor (cantilever tip height as measured from the surface) is 1.4 mm.

The electrical measurement procedure depends on two piezo-resistive strain gauges fixed to flexible cantilevers bent out-of-plane, which are deflected by the mechanical force of the moving fluid. Each micro strain gauge runs across the entire surface of the cantilever and accordingly changes its electrical resistance even at the tiniest stretching and compressing of the beam. A built-in Wheatstone bridge circuit, which implements a half-bridge strain gauge configuration, maximizes the signal transduction and provides measurable differences in voltage proportional to the deformation of the beams. Voltage signals below and above the potential difference of the Wheatstone bridge circuit in its equilibrium (offset) state are directly related to the direction of the flow.

**Figure 4:**
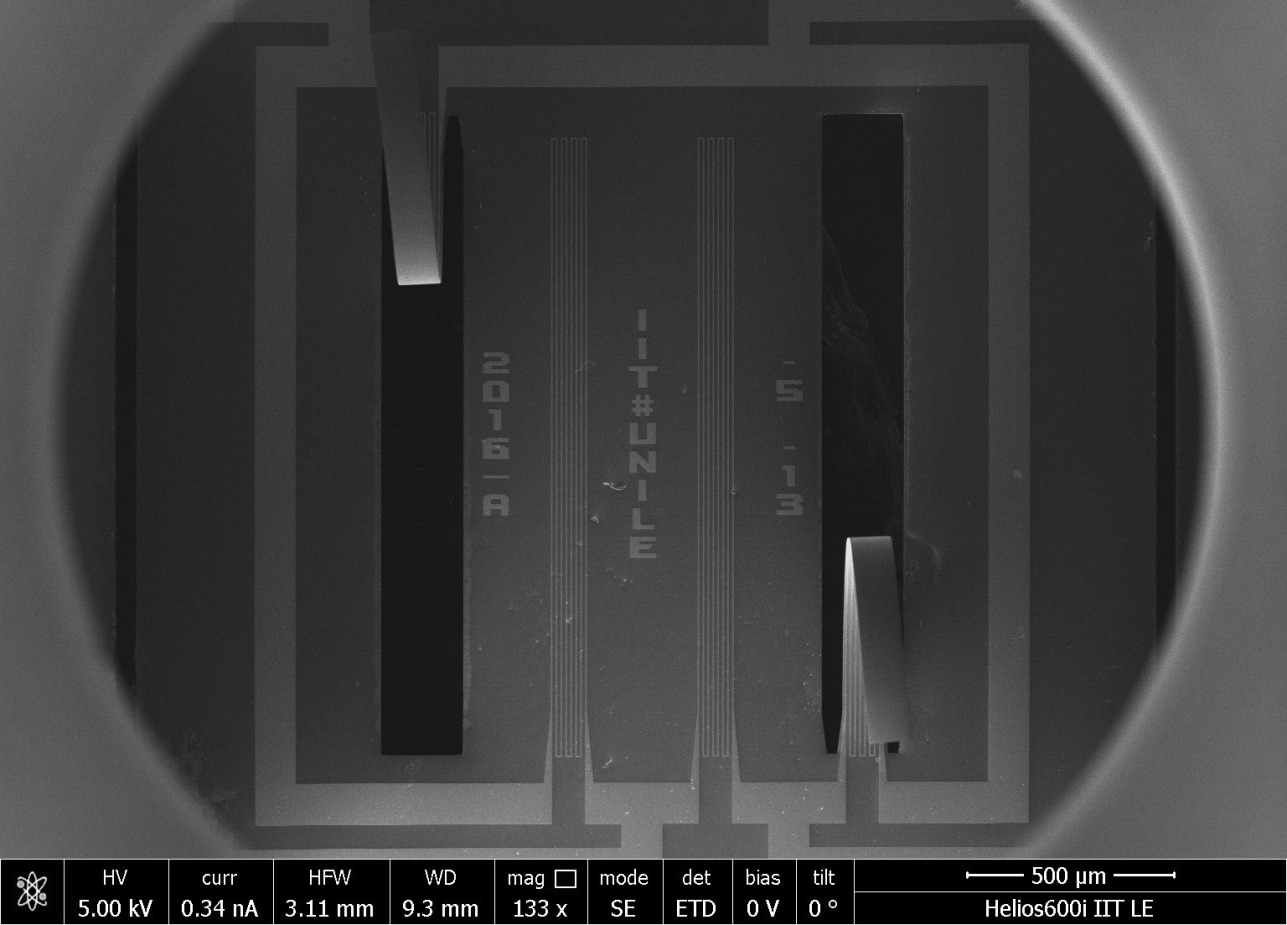
SEM picture of the antiparallel artificial hair sensor (top view). Four adjoined resistors, two static resistors in the centre and two bent out-of-plane strain gauges (variable resistors), form a half-bridge strain gauge configuration.

**Figure 5:**
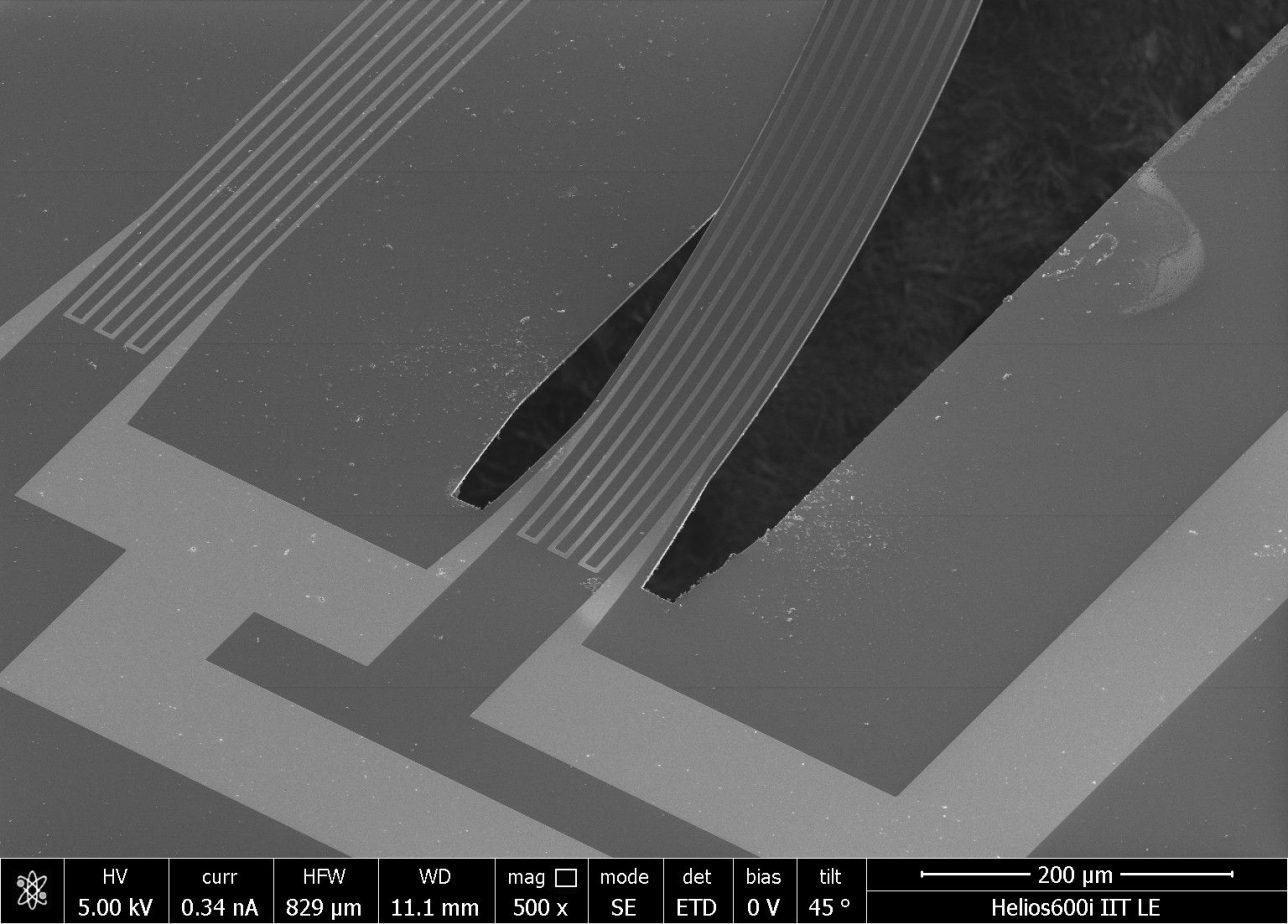
SEM picture of the cantilever hinge. The U-shaped release pattern and the micro strain gauges including connection wires are visible.

The antiparallel artificial hair sensor is equipped with two bent cantilevers and has a squared footprint of 2.5 mm^2^. The cantilevers reach approximately 1 mm tip height above the squared sensor substrate. With a substrate height of ≈400 μm, the total height of the flow sensor adds up to 1.4 mm. Each cantilever is 1.5 mm long, 100 μm wide and, depending on the thickness of the parylene layer, 2 to 4 μm thick. Four contact pads, located in each of the four corners on the sensor top side, interface power supply (excitation voltage) and voltage readout with peripheral data acquisition hardware. SEM pictures are presented in Figure 4 and Figure 5.

As shown in Figure 5 and listed in Table 2, a single strain gauge is made up of 8 strain gauge periods with a pitch of 12 μm between two neighbouring periods. A single period is 1500 μm long, 4 μm wide and has a thickness of 100 nm.

**Table 2:** Symbols, parameters and values used for the physical model of the antiparallel artificial hair sensor.

Symbol	Parameter	Value

*n*_p_	number of strain gauge periods	8
*p*_p_	pitch between two neighbouring periods (for flat and bent strain gauges)	12 μm
*l*_flat_	length of a flat strain gauge period	1500 μm
*w*_flat_, *w*_bent_	width of a strain gauge period (for flat and bent strain gauges)	4 μm
*t*_flat_	thickness of a flat strain gauge period	100 nm
ρ_NiCr_	resistivity for nichrome 80/20	1.36 × 10^−6^ Ω m^−1^
*t*_beam_	thickness of the cantilever beam	2.3 μm

**Figure 6:**
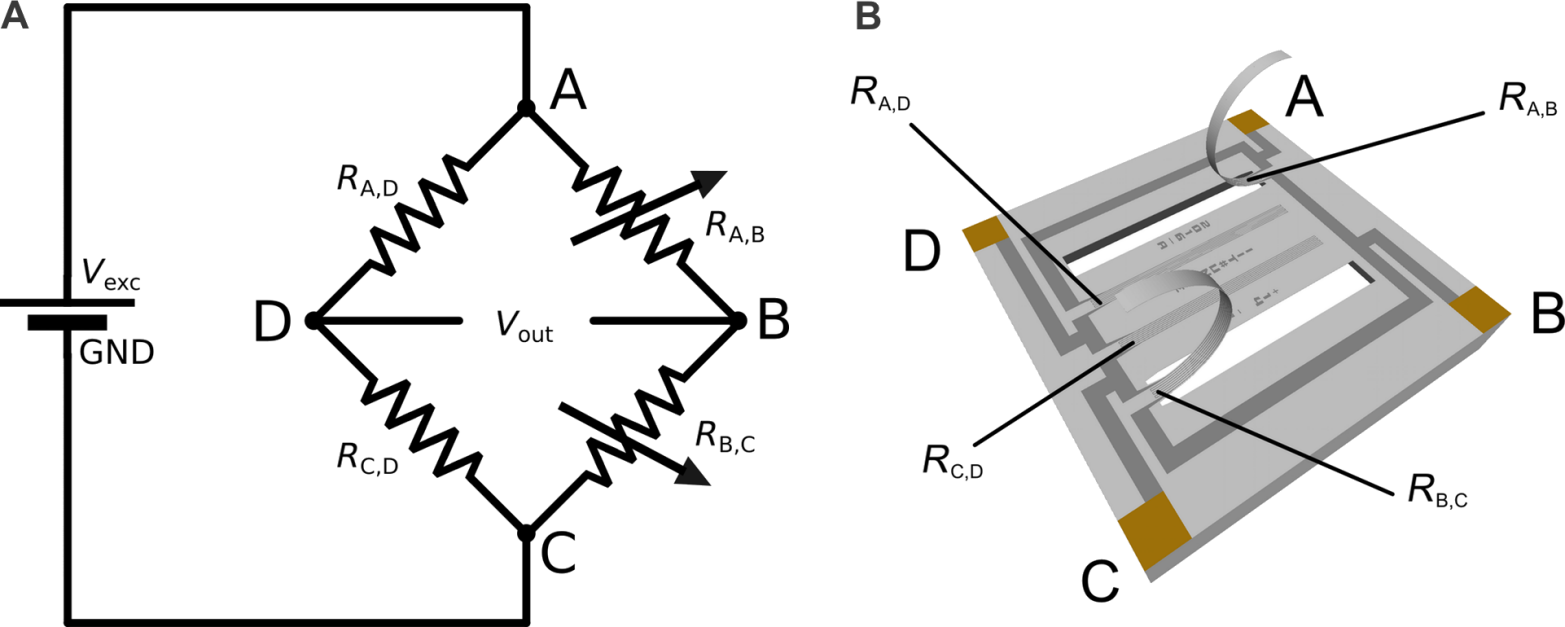
(A) Schema and (B) implementation of a Wheatstone half-bridge circuit with two static and two variable resistors (strain gauges).

The equivalent circuit resistance between two adjoined points in an unexcited Wheatstone bridge circuit, say points A and B as pictured in Figure 6, is represented by a parallel circuit of a single resistor in the one arm (*R*_A,B_) and a series of three resistors in the other arm (*R*_B,C_ + *R*_C,D_ + *R*_A,D_). In case of opposed points, say points A and C, the circuit consist of two arms with two strain gauges in series, respectively.

Table 3 provides an overview of the strain gauge resistances at rest (without power supplied to the circuit) as measured prior to any experiments. Interconnected strain gauge resistances read approximately 31 kΩ for adjoined (e.g., *R*_A,B_) and 41 kΩ for opposed (e.g., *R*_A,C_) strain gauges. This result demonstrates that the measured resistances are in accordance with the modeled resistances.

**Table 3:** Modeled and measured resistances for antiparallel artificial hair sensors.

Type	Resistors	Modeled resistances [kΩ]	Measured resistances [kΩ]

isolated, flat strain gauge	*R*_flat_	40.98	

isolated, bent strain gauge (semi-circular)	*R*_bent_	40.72	

isolated, bent strain gauge under maximal compression	*R*_bent(max)_	40.69	

interconnected, adjoined resistor	*R*_A,B_	30.73	30.66
	*R*_B,C_		31.08
	*R*_C,D_		30.66
	*R*_A,D_		30.80

interconnected, opposed resistor	*R*_A,C_	40.85	41.05
	*R*_B,D_		41.18

The change in resistance for an upwardly bent strain gauge is caused by the compressing surface strain of the curled-up cantilever. To fulfil the condition that the volume of the strain gauge is constant even under compression, an increase of the strain gauge thickness is expected, which in turn decreases the electrical resistance. For a previously described cantilever beam under maximal compression in nitrogen flow, the least possible resistance value was found to be approximately 0.07% smaller than the resistance value in resting, bent position [44]. This value is used to approximate the resistance value of the bent strain gauge under maximal compression in air flow. The theoretical voltage range is defined by the minimal and maximal possible resistance values in the circuit, which are reached in two conditions. Both conditions are met when the cantilevers undergo maximal loading, that is, maximal stretching for the one and maximal compressing for the other beam: for each of the two flow directions, one strain gauge is highly compressed (*R*_bent(max)_, flow hits the cantilever back side and causes the beam to curl up further) while the other is flat (*R*_flat_, flow hits the cantilever front side and flattens the beam).

The output voltage *V*_out_, that is, the voltage of point D relative to point B, as shown in Figure 6, is defined as

[1]
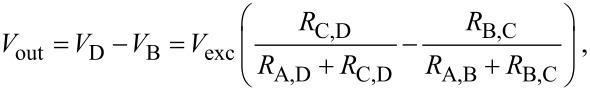


where *V*_D_ and *V*_B_ are the potentials at point D and B, respectively, and *V*_exc_ is the excitation voltage. As indicated in Figure 6, the variable resistors (strain gauges) are located in the right arm, that is *R*_A,B_ and *R*_B,C_. With an excitation voltage of 3.3 V, the offset (resting), minimal and maximal voltage signals for the antiparallel artificial hair sensors approximate:

[2]
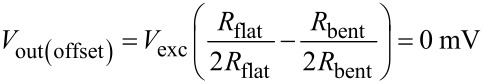


[3]
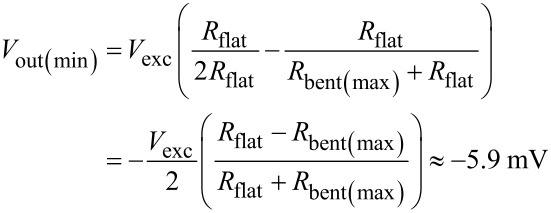


[4]
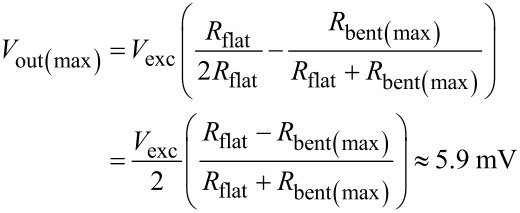


Following the same mathematical procedure, the quarter-bridge configuration of a single artificial hair sensor (with strain gauge *R*_A,B_) generates output signals that range between *V*_out(min)_ ≈ −5.9 mV and *V*_out(max)_ = 0 mV, with resting voltage *V*_out(offset)_ ≈ −5.3 mV.

## Results

### Bidirectional air flow

**Figure 7:**
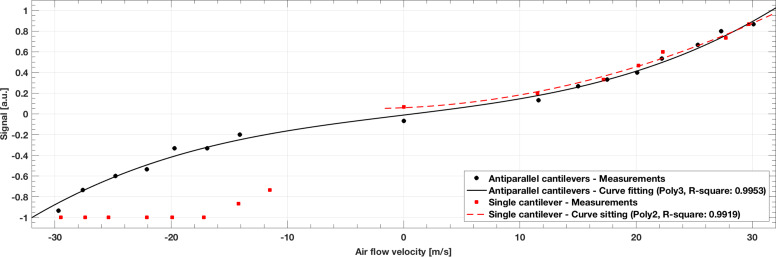
Calibration curve for the single and antiparallel artificial hair sensors. Dots indicate measurement values and the lines represent curve fittings for visual guidance. For the single cantilever, a second-order polynomial fit (R-square: 0.9919) was done for the forward air flow direction. It reached its saturation limit for backward air flow at −17.2 m s^−1^. The third-order polynomial fit for the antiparallel cantilevers (R-square: 0.9953) proposes an axially symmetrical sensitivity of 40 μV/(m s^−1^) for the lower air flow velocity range (between ±10–20 m s^−1^) and 80 μV/(m s^−1^) for a higher air flow velocity range (between ±20–32 m s^−1^).

Figure 7 presents the experimental results for the bidirectional air flow measurements. As both flow sensor variants differed in offset voltage and output range, the signal amplitudes were normalized. The dots indicate measurement values and the lines represent polynomial curve fittings for visual guidance. For the antiparallel artificial hair sensors, the third-order fit (R-square: 0.9953) proposes an axially symmetrical sensitivity of 40 μV/(m s^−1^) for the lower air flow velocity range (between ±10–20 m s^−1^) and 80 μV/(m s^−1^) for a higher air flow velocity range (between ±20–32 m s^−1^). The absolute measurement values ranged between 9.6 mV and 12.6 mV around the offset voltage of 11 mV for the antiparallel artificial hair sensors. While the single cantilever showed a comparable sensitivity for the forward air flow direction, it reached its saturation limit at −17.2 m s^−1^ for the backward direction, resulting in an asymmetrical response behaviour.

### Influence of temperature on micro strain gauges

**Figure 8:**
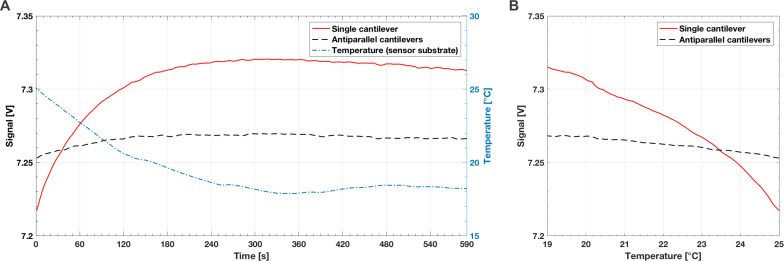
(A) Influence of temperature changes on micro strain gauges as a function of time. The offset voltage of the single cantilever drifted around 100 mV, while the antiparallel cantilevers drifted only around 15 mV. (B) Temperature offset curve for the single and antiparallel artificial hair sensors.

As shown in Figure 8, the offset voltage of the Wheatstone quarter-bridge circuit (single cantilever) drifted around 100 mV, from 7.22 V up to 7.32 V after cooling down the sensor unit from 25 °C to 19 °C. This drift suggests that the sudden temperature decrease influenced the offset voltage for the single cantilever sensor. In the subsequent warming up phase (data not shown), the output dropped back to its offset voltage (7.22 V). Accommodating for signal amplification, the actual drift was 1 mV. The temperature experiments were repeated for the antiparallel artificial hair sensors. The results in Figure 8 show that the Wheatstone half-bridge circuit with two bent strain gauges reduced the effects of temperature changes. A perfectly balanced half-bridge circuit with two identical resistors in both arms would fully compensate for temperature changes. Due to variation in the resistances (see Table 3), the half-bridge circuit was not perfectly balanced and a drift of 15 mV was measured. Accommodating for signal amplification, the actual drift was 0.15 mV. The artificial hair sensors did not generate heat, which suggests that they changed their temperature with the environment only.

### Multidirectional air flow

**Figure 9:**
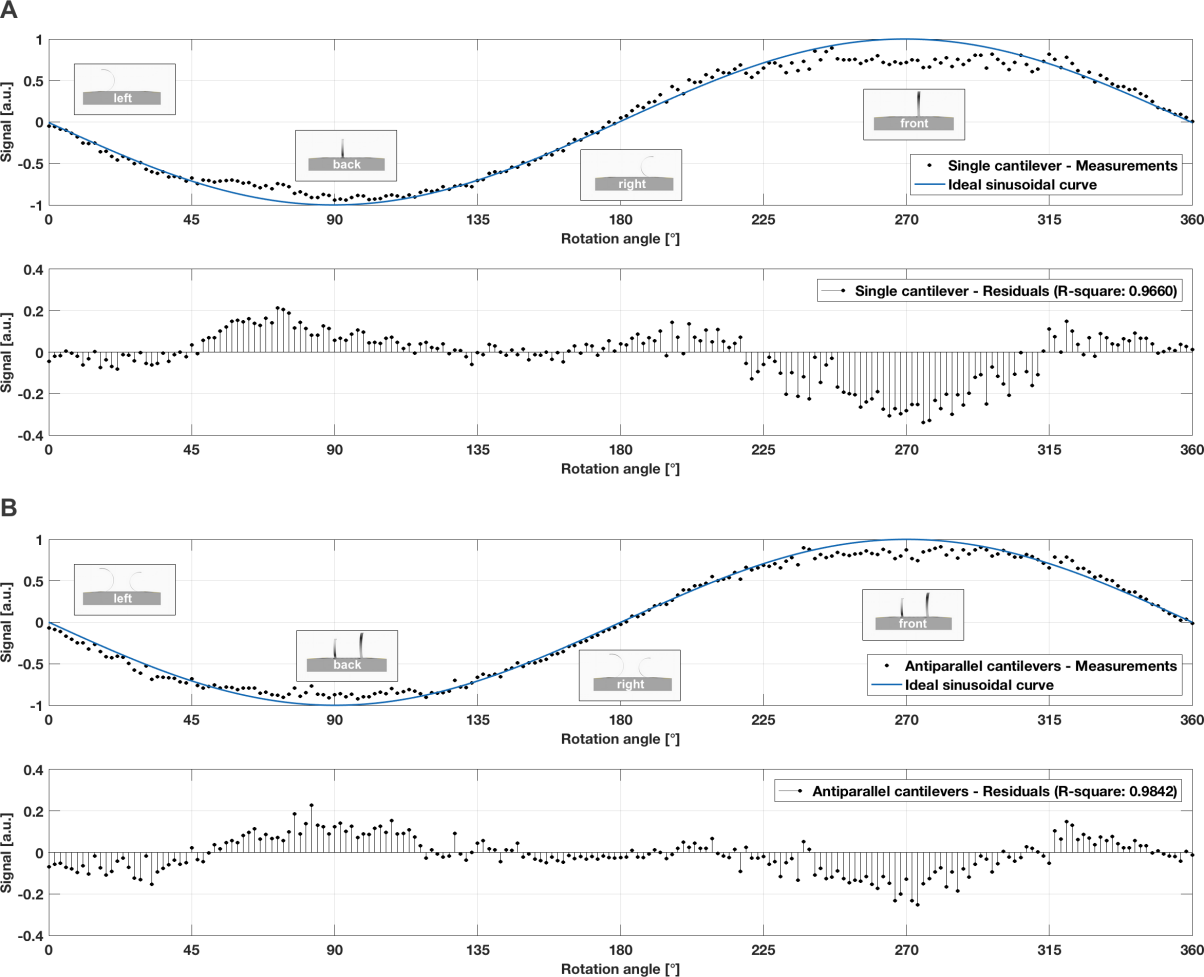
Response behaviour of the (A) single and (B) antiparallel artificial hair sensor to multidirectional air flow. The first (upper) plots compare the measured output voltage of the Wheatstone bridge (black dots) with the expected ideal sinusoidal curve (blue curve), whereas the second (lower) plots present the residuals of the ideal signal. Data (average mean of 30,000 measurement values) is shown as a function of rotation angle. Signal amplitude was normalized.

Figure 9 present the average mean of 30,000 measurement values for the single and antiparallel hair sensors in multidirectional air flow. The sensors were gradually rotated in steps of 1.8° until a full cycle (360°) was reached. The first (upper) plot for each sensor variant compares the measured output voltage of the Wheatstone bridge (black dots) with the expected ideal sinusoidal curve (blue curve), whereas the second (lower) plot presents the residuals of the ideal signal. The data is shown as a function of rotation angle. As both flow sensor variants differed in offset voltage and output range, signal amplitudes were normalized. The overlaid graphics indicate the sensor orientation to the flow direction, which points directly into the plot (*z*-axis).

Both flow sensors gave a consistent, sinusoidal response to the multidirectional air flow conditions, which was expected, as drag force on the bent cantilevers is a function of flow direction (or rotation angle in the presented experiment). When a cantilever is rotated by angle α, the expected signal amplitude is *A*_α_ = *A*_max_sin(α), where *A*_max_ is the signal amplitude for the rotation angle of best directional sensitivity, namely 90° and 270° in case of the two flow sensor variants during the experiments. The expected amplitude for a rotation angle α = 45° corresponds to approximately 71% of the maximal signal amplitude. Both flow sensor variants showed the expected signal output (±0.7 for the normalized data) for the rotation angles 45°, 135°, 225°, and 315°. However, the antiparallel artificial hair sensors generated a more precise sinusoidal response, as indicated by the higher R-square value (0.9842 for the antiparallel configuration vs 0.9660 for the single cantilever). In forward flow (270°), the single sensor reached its saturation limitation, flattening at 67% of the ideal sinusoidal curve whereas the antiparallel cantilevers saturated at 75%, as indicated by the residuals in Figure 9. Although both sensor variants showed a signal saturation for 90° and 270° (where the deviation from the ideal sinusoidal signal increased), the antiparallel strain gauges generated a higher signal in these particular positions. This behaviour can be explained, as one strain gauge is bent down while the other is curved up, which represents two changing resistances in opposite directions, one getting smaller and one getting bigger. That amplifies the electrical effect in the Wheatstone bridge circuit, which in turn supports higher ranges in the output voltage. Therefore, antiparallel cantilevers resulted in improved directional sensitivity and sinusoidal response to flow angle. While the antiparallel cantilevers provided maximal signal for rotation angles of best directional sensitivity (90° and 270°), it inhibited voltage output for the two other main directions, where air flow hit the sensors from the side (0° and 180° in our experiment). In these two conditions, where the cantilever surface is minimal and aerodynamic forces are hardly exerted on the cantilever beam, the cantilevers oscillated around their resting position. Even under laminar conditions, the bent cantilever is a flexible bluff body that generates periodic turbulence, which causes oscillation around its resting position.

**Figure 10:**
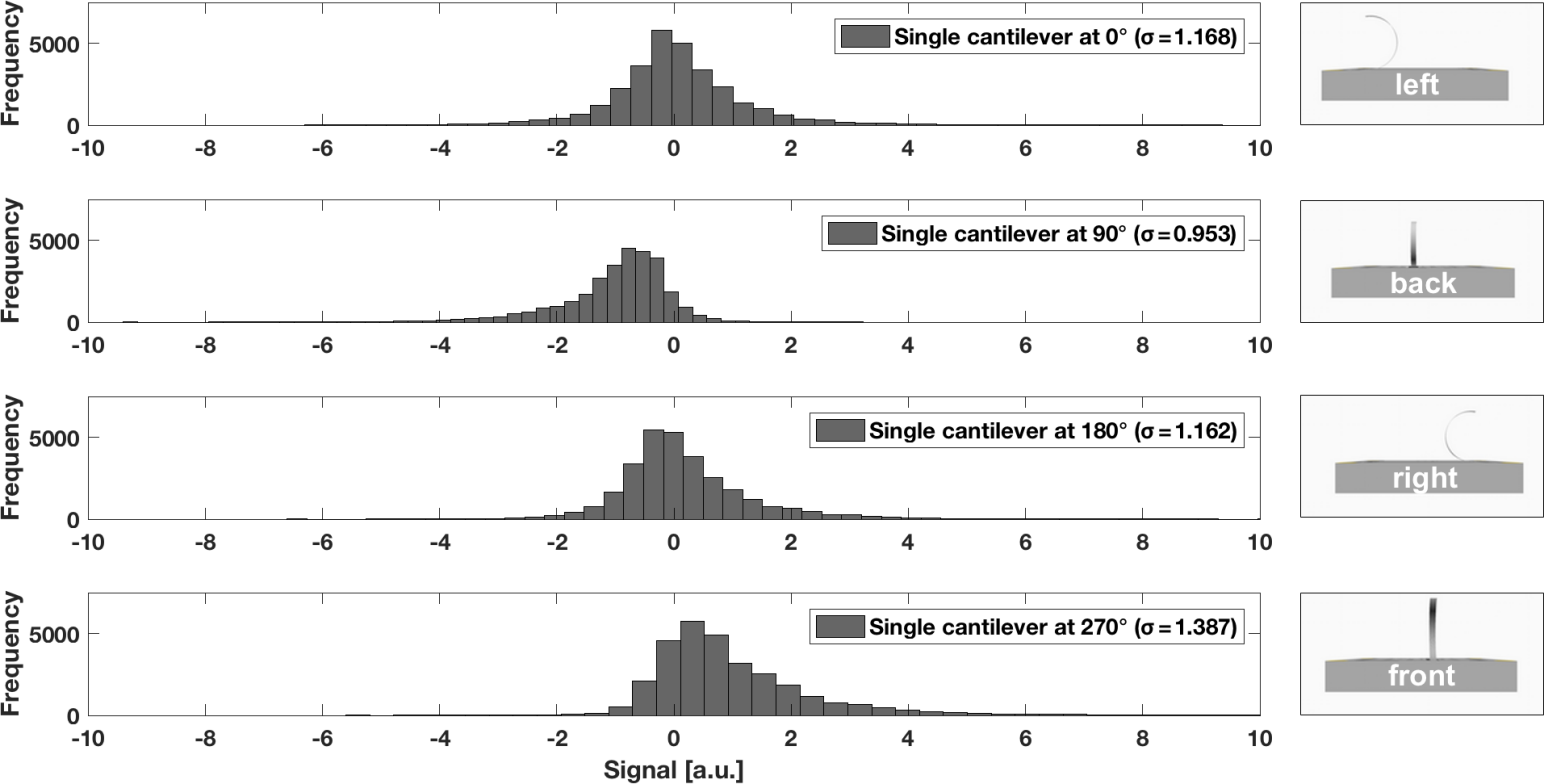
Average mean distribution of 30,000 measurement values for the single cantilever at rotation angles 0°, 90°, 180°, and 270°. Standard deviations (σ) are listed in the legend.

**Figure 11:**
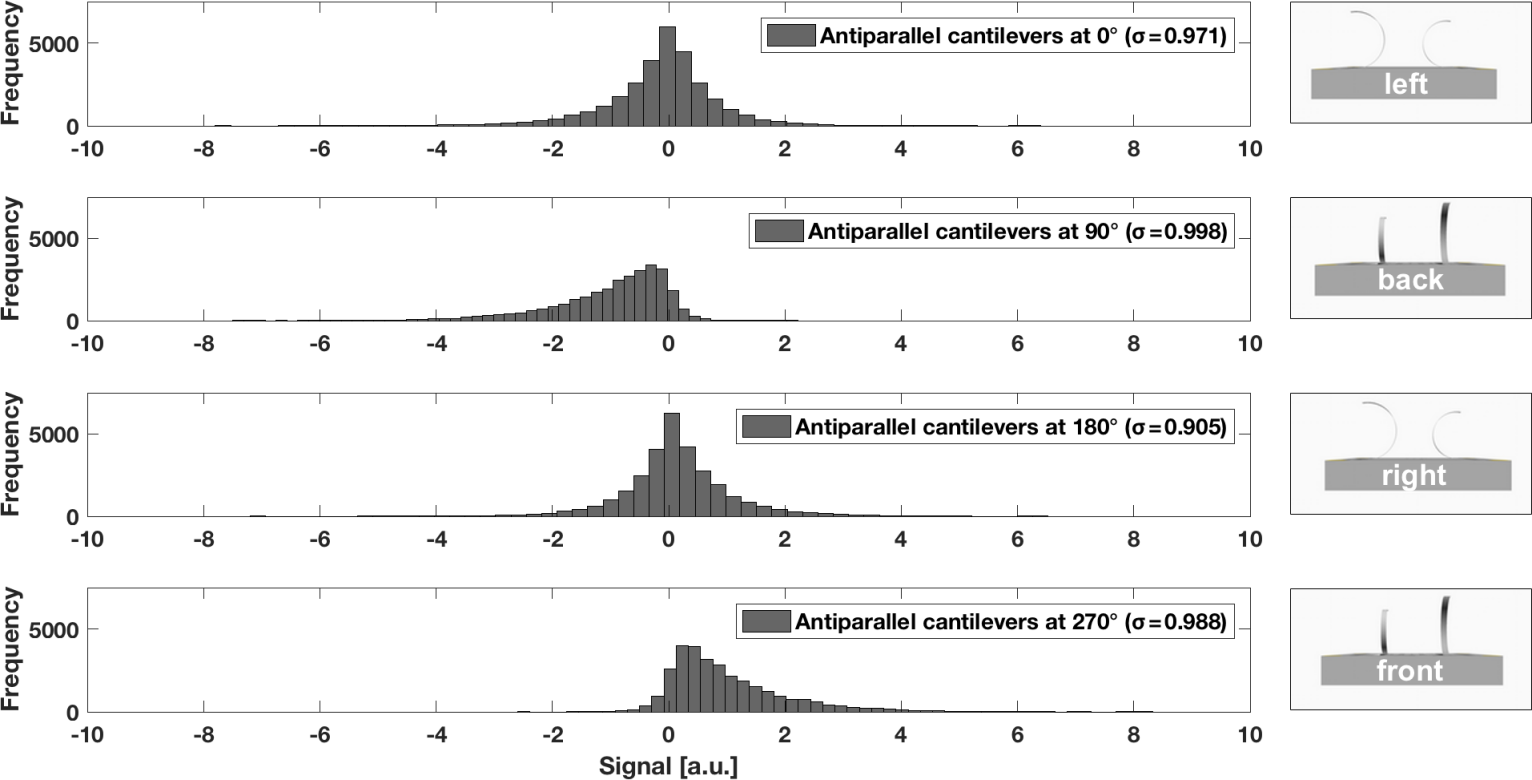
Average mean distribution of 30,000 measurement values for the antiparallel artificial hair sensors at rotation angles 0°, 90°, 180°, and 270°. Standard deviations (σ) are listed in the legend.

The histograms in Figure 10 and Figure 11 present the average mean of 30,000 measurement values at rotation angles 0°, 90°, 180°, and 270°. As expected, the cantilevers oscillated around the resting position when the air flow hit both sensor variants from the side (0° and 180°). The single cantilever revealed a more skewed, broader distribution (Figure 10), whereas the antiparallel cantilevers showed a more symmetric, narrower distribution (Figure 11). The same behaviour was identified for the two rotation angles of best directional sensitivity, that is 90° and 270°. The antiparallel artificial hair sensors were less noisy and showed a statistical symmetry, as indicated by the similarly shaped but mirror-inverted distribution for the forward and backward air flow directions. This fact can also be explained when comparing the standard deviations for the directions of best sensitivity. While the single cantilever showed a difference of ≈0.43 between the two directions, that is 1.387 for the forward (cantilever flattened down at 270°) and 0.953 for the backward air flow direction (cantilever curled up at 90°), the antiparallel cantilevers showed a more consistent symmetry for these rotation angles (0.01 difference). The bigger difference in the standard deviation suggests that the single cantilever generated a noisier signal for the 270° (cantilever flattened down) when compared to the 90° rotation (cantilever curled up), which supports the assumption that the cantilever oscillated less in the compressed state, because it is less flexible in the compressed state.

## Discussion

In this work, antiparallel artificial hair sensors, coupled as a flow sensor with bidirectional sensitivity, were fabricated and fully characterized. The novel sensor design and arrangement was calibrated and stimulated by air flow in multiple directions. Sensitivity to temperature changes was measured. All experiments and measurements were compared with a single hair sensor reference model with equal cantilever dimensions and material composition.

The conducted experiments under bidirectional air flow conditions demonstrated that the antiparallel artificial hair sensors exhibit an axially symmetrical sensitivity range between 40 μV/(m s^−1^) for the lower air flow velocity range (between ±[10–20] m s^−1^) and 80 μV/(m s^−1^) for a higher air flow velocity range (between ±[20–32] m s^−1^). Compared to the cantilever based flow sensor described by Du et al. [40], which provided an asymmetrical best sensitivity of 60 μV/(m s^−1^) at 18 m s^−1^, the presented antiparallel cantilevers demonstrated an improved sensitivity for air flow velocities higher than 20 m s^−1^. Whereas the presented single cantilever reference model showed a saturation limit at −17.2 m s^−1^ (for a curled-up cantilever in backward air flow direction), the antiparallel configuration provided sensitivity to both air flow directions, overcoming the saturation limit by having the second cantilever extended out into the flow. The theoretical output voltage was estimated to fluctuate between ±5.9 mV around the offset voltage for an excitation voltage of 3.3 V. The measurement values ranged between 9.6 mV and 12.6 mV around the offset voltage of 11 mV for the antiparallel artificial hair sensor. The mismatch between the measured (3 mV) and the modeled (11.8 mV) output range can be explained by the fact that the maximal air flow velocity of the wind tunnel (32 m s^−1^) did not generate enough drag force to flatten the downwind cantilever completely. In our previous work, we obtained signal saturation in air at about 40 m s^−1^ for a comparable but not mechanically identical cantilever [47]. Other authors described detection limits for comparable cantilevers as 45 m s^−1^ [38], which confirms the suggested explanation for the mismatch in the measured output voltage range.

The presented strain gauges were subject to temperature changes, which are caused by two effects. The first is a difference in convective cooling due to the perpendicular air flow over the strain gauges on the extended cantilevers versus the parallel flow over the gauges on the sensor substrate. The second effect is the difference in heat capacity of the thin cantilever versus that of the much more massive sensor substrate, which affects both the heat flow by conduction and the temperature change of the substrate itself. To investigate the influence of temperature on the micro strain gauges, the presented artificial hair sensors were subjected to a sudden decrease in temperature, from 25 °C down to 19 °C in about 4 minutes. As a consequence, the offset voltage of the single cantilever sensor drifted by 1 mV, whereas the antiparallel cantilevers showed a smaller influence of the temperature decrease, a drift of 0.15 mV. The Wheatstone half-bridge circuit with two bent strain gauges did not compensate for the temperature change completely, as it was not perfectly balanced (see the variation in the resistances in Table 3). Nevertheless, it reduced the influence of temperature by a factor of 6.7 when compared to the single cantilever sensor, as shown in Figure 8. In previously published work, various thermal effects on piezoresistors such as friction, self-heating and convection were described. Du et al. proposed an additional temperature resistance that could better compensate for temperature changes [41]. Other authors fabricated temperature compensation circuits together with the strain gauges on a single chip, as described by Ozaki et al. [50]. Their proposed (but not discussed) approach for on-chip temperature compensation was a Wheatstone full-bridge configuration with four active strain gauges. In general, temperature compensation is required when bent out-of-plane cantilevers operate in large air flow velocity ranges, where wind-induced convection and in turn temperature changes are likely to occur.

Drag force on the bent cantilevers is a function of flow direction (or rotation angle) and therefore implies a sinusoidal response behaviour, which was already demonstrated by previously published work. The flow sensor described by Wang et al. [39] revealed a sinusoidal relationship between air flow angle and resistance variation, with the largest variation for the downwind, flattened cantilever and the least variation for the upwind, curled up cantilever. The resistance variations of a cantilever positioned perpendicular to the air flow direction was almost equal to zero. Ozaki et al. [50] and Du et al. [41] used a Wheatstone half-bridge circuit to convert the resistance variations into a related output voltage, which demonstrated the expected sinusoidal relationship between air flow angle and output voltage signal. In our work, as presented in Figure 9, the performed measurements under multidirectional air flow conditions matched the expected values, as both flow sensors gave a sinusoidal response to the multidirectional air flow. The single sensor reached its saturation limitation in forward flow at 67% of the ideal sinusoidal curve whereas the antiparallel cantilevers saturated later at 75%. When the air flow hit both sensor variants from the side (cantilevers oscillated around the resting position), antiparallel cantilevers showed a more symmetric, narrower distribution. For forward and backward air flow direction, the antiparallel artificial hair sensors were less noisy and showed a statistical symmetry, as indicated in the histograms in Figure 10 and Figure 11. This result implies that the antiparallel artificial hair sensors generated less noise while oscillating around their offset voltage and provided higher sensitivity over the entire multidirectional measurement range.

The presented novel antiparallel flow sensor design was inspired by the neuromasts found in the lateral line of fish, neuromast sensors which have a directional sensitivity (determined by an axis of orientation of the individual hair cells) and morphological polarity [1]. As in the biological model, where hair cells within the same neuromast assume an antiparallel bending orientation with respect to each other, the presented antiparallel artificial hair sensors also demonstrated an axis of sensitivity or best direction.

## Conclusion

As indicated by the two presented artificial hair sensors, a sinusoidal response to flow stimulus has been measured, mimicking the cosine-like response function of the afferent nerves of the hair cells. The clear response angle for the antiparallel artificial hair sensors creates a unique sensor with improved directional sensitivity. Its response to oscillatory flow has yet to be determined; however, as one cilia flattens, the other remains extended out into the flow and may therefore be capable of responding to additional flow stimuli. Despite the fact that the direction of flow cannot be measured by the antiparallel cantilever configuration in principle (an orthogonal configuration would have to be formed to directly measure two-dimensional flow direction, which we are planning to build in the upcoming year), it demonstrated improved bidirectional sensitivity to flow sensing with curved artificial hair sensors.

In the short term, the presented sensor platform will be investigated in liquids as well, once the sensors are embedded in a waterproof MEMS package. For future applications, we are targeting automotive, robotics, automation and air conditioning applications with variable air flow speeds, ranging between a few and 40 m s^−1^. A first real-world case study with our flow sensor platform was designed and conducted by the Institute of Air Handling and Refrigeration in Dresden (Germany), which aimed at detecting angular momentum in industrial air ducts for controlling the speed of contra-rotating axial fans. A given technical problem of axial fans is the emergence of unavoidable swirl in the wake flow with high peripheral speeds. While contra-rotating axial fans already reduce the swirl in the wake flow [51], detecting angular momentum in real-time using our flow sensor platform could possibly further reduce mechanically unfavourable flow dynamics in the wake.

When compared to flow sensors which are based on the same electrical measurement procedure (piezoresistive, strain gauge) and mechanical structure (bent cantilever), the achieved sensitivity of our device is comparable to previously published work by other authors. Our presented research focussed on how to improve the response behaviour of bent piezoresistive cantilever structures. The successive improvement of the bidirectional sensitivity, that is, improved temperature compensation, decreased noise generation and symmetrical response behaviour, can be considered the primary result of the presented research. The specific advantage of our design is that we can measure flow speeds directly instead of having to infer them from Bernoulli assumptions based on pressure differences.

## Experimental

### Sensor fabrication

During the fabrication process, thin silicon nitride/silicon layers are stacked together on a silicon wafer substrate and precisely shaped into the desired cantilever geometry by performing an etching process. In the following, we describe the MEMS fabrication process for the stress-driven artificial hair sensor. The fabrication process is subdivided into the following main steps:

- **Depositing functional material layers:** The flow sensor is based on a silicon-on-insulator (SOI) substrate which is made up of a 400 μm silicon wafer, a 2 μm thick silicon dioxide (SiO_2_) insulation layer, and a 2 μm silicon device layer. The SOI substrate is coated with a 300 nm SiN layer on the bottom and top side.
- **Depositing piezoresistors and contact pads:** The actual fabrication process starts with a physical thermal evaporation of four nickel–chrome (NiCr) 80/20 (80% nickel, 20% chrome) piezoresistors, which are 100 nm thick and distributed along the full length of the cantilever beam. In a subsequent deposition step, four 150 nm thick gold (Au) contact pads are deposited.
- **Defining the cantilever U-shapes:** A trifluoromethane (CHF_3_) based inductively coupled plasma (ICP) dry etching step at the substrate top side was performed to generate the U-shaped release patterns for two cantilever beams. The top side SiN layer is patterned and etching is stopped at the silicon device layer (300 nm etching depth).
- **Releasing the cantilevers:** In this manufacturing step, the generated U-shaped patterns are used to release the cantilevers. The ICP etching in the previous step opens two U-shaped windows to the silicon device layer. A second ICP etching process (as described in the previous step) of the SiN layer is performed at the wafer back side to open two apertures to the silicon bulk substrate underneath the cantilever beams (300 nm etching depth). Subsequently, anisotropic back side wet etching with a potassium hydroxide (KOH) 28% solution at 85 °C creates two cavities underneath the cantilever beams (400 μm etching depth). The SiO_2_ insulating layer acts as an etching barrier. Next, a hydrofluoric acid (HF) back side wet etching step removes the SiO_2_ layer (2 μm etching depth). By performing a last KOH top side wet etching, the exposed (U-shaped) silicon device layer around the cantilevers is removed (2 μm etching depth). Due to the residual stress in the material, the released SiN/Si bilayer bends out of the plane upon release [37]. The resulting cantilever thickness is 2.3 μm.
- **MEMS packaging:** After wire bonding the contact pads to an integrated circuit socket adapter (W13028RC, Winslow ADAPTICs), chemical vapour deposition of parylene at room temperature is performed which adds a conformal 2 μm encapsulation layer to all sides of the cantilever beams as well as the flow sensor substrate (including circuitry). This process tunes the mechanical properties (flexural stiffness) of the artificial hair sensor, as described in [46].

### Experimental methodology

Three different experiments were designed and conducted to investigate the performance of both stress-driven hair sensor variants to flow stimuli:

1. Both variants were compared and calibrated under bidirectional air flow conditions, that is two air flow directions with reference to the two bending axes of the cantilevers.
2. Both variants were subjected to thermal variations to compare the influence of temperature on the micro strain gauges.
3. Both variants were compared under multidirectional air flow, that is flow sensors were gradually rotated until a full cycle was achieved (360°).

#### Bidirectional air flow

The facility used for the first experimental research was a subsonic wind tunnel, operated by the Department of Civil and Industrial Engineering at University of Pisa. The closed-return Goettingen type wind tunnel had an open (round) test section of 1.1 m in diameter, 1.4 m in length, and was characterized by a free-stream turbulence level of 0.9%. A computer-controlled traversing rig positioned the artificial hair sensors at the centre of the wind tunnel. In our measurements, air flow velocities between 12 and 32 m s^−1^ were generated and tracked by a calibrated pitot tube and thermoresistance based measuring equipment. In the wind tunnel, Reynolds numbers range between *R*_e_ ≈ 67 (for 10 m s^−1^) and *R*_e_ ≈ 213 (for 32 m s^−1^), suggesting steady flow conditions in the wind tunnel at the cantilever tip. To investigate the electrical behaviour under bidirectional air flow conditions, two different experiments were performed with flow sensors aligned in forward and backward flow direction with reference to the two bending axes of the cantilevers. For both experiments, the velocity range between 12 and 32 m s^−1^ was applied to measure the electrical response of the flow sensors. During the experiments, the built-in Wheatstone bridge circuit was excited with *V*_exc_ = +3.3 V DC, while its voltage output was connected to a benchtop digital multimeter (Agilent 34405A).

#### Influence of temperature on micro strain gauges

As electrical resistivity of metals is temperature-dependent, a drift in the offset voltage may occur while the flow sensor is in operation. To compare the influence of temperature on air flow measurements, both flow sensor variants (single and antiparallel artificial hair sensors) were subjected to a sudden decrease in temperature. A cylindrical ice block (6 cm diameter and 7 cm height) was cooled down to −11 °C and situated directly beneath the flow sensor for 10 minutes, while the offset voltage of the Wheatstone bridge was recorded. The built-in Wheatstone bridge circuit was excited with *V*_exc_ = +5.0 V DC. To eliminate additional highly complex thermodynamics, measurements were performed without air flow. A high-resolution infrared camera (FLIR, SC600-series, IR lens *f* = 13.1 mm), positioned in 20 cm distance, was used to track the temperature changes at the sensor substrate.

#### Multidirectional air flow

A second wind tunnel at the Rhine-Waal University of Applied Sciences was used to conduct experiments under multidirectional air flow conditions. The rectangular wind tunnel was 1.5 m long, 0.40 m wide, 0.55 m high and provided air flow velocity of 10.5 m s^−1^, as locally measured with a commercial hot wire anemometer sensor (Trotec TA300). To generate multidirectional air flow, a sensor mount was fixed to a motor shaft which was connected to a computer-controlled stepper motor (Astrosyn MY180). The flow sensors were positioned in the horizontal centre of the cross-sectional area of the wind tunnel, 0.35 m above the bottom, and at a distance of 1 m to the flow inlet. While gradually rotating the flow sensor in steps of 1.8°, voltage output was amplified (100× amplification factor), converted analogue-to-digital with 14 bits precision (NI USB-6009), recorded and post-processed with a commercial software (MATLAB). In each of the 200 positions (for a full cycle of 360 degrees), 10,000 samples were taken with a sampling rate of 1 kHz. The Wheatstone bridge circuit was excited with *V*_exc_ = +5.0 V DC. The experiment was repeated three times for both artificial hair sensors.
